# Comparison of Extended-Spectrum Beta-Lactamase-Producing *Escherichia coli* Isolates From Rooks (*Corvus frugilegus*) and Contemporary Human-Derived Strains: A One Health Perspective

**DOI:** 10.3389/fmicb.2021.785411

**Published:** 2022-01-13

**Authors:** Bálint József Nagy, Bence Balázs, Isma Benmazouz, Péter Gyüre, László Kövér, Eszter Kaszab, Krisztina Bali, Ádám Lovas-Kiss, Ivelina Damjanova, László Majoros, Ákos Tóth, Krisztián Bányai, Gábor Kardos

**Affiliations:** ^1^Department of Medical Microbiology, Faculty of Medicine, University of Debrecen, Debrecen, Hungary; ^2^Doctoral School of Pharmaceutical Sciences, University of Debrecen, Debrecen, Hungary; ^3^Department of Nature Conservation, Zoology and Game Management, Faculty of Agricultural and Food Sciences and Environmental Management, University of Debrecen, Debrecen, Hungary; ^4^Institute for Veterinary Medical Research, Budapest, Hungary; ^5^Department for Tisza River Research, Centre for Ecological Research–DRI, Hungarian Academy of Sciences, Budapest, Hungary; ^6^National Public Health Center, Budapest, Hungary; ^7^Department of Pharmacology and Toxicology, University of Veterinary Medicine Budapest, Budapest, Hungary

**Keywords:** ESBL carriage, *E. coli* ST131, *E. coli* ST162, *E. coli* ST744, long-distance dispersal, bird migration, CTX-M-55

## Abstract

During winter, a large number of rooks gather and defecate at the park of a university clinic. We investigated the prevalence of extended-spectrum beta-lactamase (ESBL)–producing *Escherichia coli* in these birds and compared recovered isolates with contemporary human isolates. In 2016, fecal samples were collected from 112 trap-captured rooks and investigated for presence of ESBL producers using eosin methylene blue agar supplemented by 2 mg/L cefotaxime; 2,455 contemporary human fecal samples of patients of the clinics sent for routine culturing were tested similarly. In addition, 42 ESBL-producing *E. coli* isolates collected during the same period from inpatients were also studied. ESBL genes were sought for by PCR and were characterized by sequencing; *E. coli* ST131 clones were identified. Epidemiological relatedness was determined by pulsed-field gel electrophoresis and confirmed using whole genome sequencing in selected cases. Thirty-seven (33%) of sampled rooks and 42 (1.7%) of human stools yielded ESBL-producing *E coli*. Dominant genes were *bla*_CTX–M–55_ and *bla*_CTX–M–27_ in corvid, *bla*_CTX–M–15_ and *bla*_CTX–M–27_ in human isolates. ST162 was common among rooks. Two rook-derived *E. coli* belonged to ST131 C1-M27, which was also predominant (10/42) among human fecal and (15/42) human clinical isolates. Another potential link between rooks and humans was a single ST744 rook isolate grouped with one human fecal and three clinical isolates. Despite possible contact, genotypes shared between rooks and humans were rare. Thus, rooks are important as long-distance vectors and reservoirs of ESBL-producing *E. coli* rather than direct sources of infections to humans in our setting.

## Introduction

Antibiotic resistance is a global problem impacting both human and animal health. The One Health concept sets forth that the health of people, animals, and the environment is interconnected, which fully applies to antibiotic resistance as well, as exemplified by the relationship between avoparcin usage and the spread of vancomycin-resistant *Enterococci* in Europe (Bager et al., 1997). Besides spread of resistant strains, gene flow between bacteria of human and animal origin drives the dissemination of resistance genes (Graham et al., 2019). Zoonotic or environmental reservoirs served as sources for emerging resistance genes, e.g., *Kluyvera* spp. as source for *bla*_CTX–M_ genes, *Shewanella algae* as source for *bla*_OXA–48_, or *Acinetobacter radioresistens* as source for *bla*_OXA–23–like_ genes (Livermore et al., 2007; Poirel et al., 2008; Tacão et al., 2018). Resistant bacteria can spread between humans and their households involving their companion animals, and the environment and wildlife. International travel, trade of animal food products, and wildlife migration further contribute to the global dissemination of antibiotic resistance (Guenther et al., 2011; Hussain et al., 2017, 2019; Zendri et al., 2020).

*Escherichia coli* is a characteristic example linking One Health and antibiotic resistance, being a frequent and abundant member of both human and animal gut microbiome as well as an important pathogen of humans and animals. The massive usage of antibiotics both in human medicine and animal industry led to contamination of natural environments with antimicrobials, antibiotic resistance genes, and resistant human pathogens (Graham et al., 2014, 2019). Wildlife living in contaminated habitats such as landfills, wastewater, sewage sludge of farms, or exposed directly to feces from livestock and companion animals can acquire resistant bacteria or resistance genes (Graham et al., 2014, 2019). These animals, particularly the highly mobile species, may scatter the resistant bacteria. Wild birds were shown to carry antibiotic resistant bacteria; typical carriers are crows (Loncaric et al., 2013; Jamborova et al., 2015) and gulls (Báez et al., 2015; Zendri et al., 2020), which often utilize human waste as food source. These birds are frequently urbanized, and their droppings pollute the cities, potentially reintroducing strains into the human environment. Because of their migration and/or vagrant behavior, these birds may serve as reservoirs and long-distance vectors both for antibiotic-resistant strains and antibiotic resistance genes (Wang et al., 2017).

Our aim was to investigate the prevalence of ESBL-producing *E. coli* carried by rooks (*Corvus frugilegus* ssp. *frugilegus*, Linnaeus 1758) gathering in a university clinic and to compare these isolates with contemporary and geographically related human-derived isolates.

## Materials and Methods

### Samples and Bacterial Isolates

Cloacal swabs were taken from 112 trap-captured rooks wintering in a suburban environment close to the clinical campus of the University of Debrecen between October 2016 and March 2017. The trapping and capturing process was conducted as previously described (Kövér et al., 2018); recapturing did not occur. In parallel, we screened all 2,455 contemporary human fecal samples of the patients of the university clinics sent for routine fecal culture during the study period to assess human asymptomatic fecal carriage of ESBL-producing bacteria using the same culture methodology. Third-generation cephalosporin (3GC)–resistant isolates were recovered using eosin–methylene blue media supplemented with 2 mg/L cefotaxime. One to three colonies per different morphologies were processed further from each sample and identified by matrix-assisted laser desorption ionization (MALDI)–time of flight (TOF) mass spectrometry (Bruker, Bremen, Germany). We also characterized 42 contemporary extended-spectrum beta-lactamase (ESBL)–producing *E. coli* isolates from various samples of inpatients of the university clinics sent for microbiological diagnostic purposes for comparison with isolates carried by rooks and humans. Production of ESBL was examined by double-disk synergy test using cefotaxime, ceftazidime, and cefepime. Susceptibility to ertapenem, ciprofloxacin, trimethoprim–sulfamethoxazole, amikacin, gentamicin, and tobramycin was determined by disk diffusion method following EUCAST guidelines.

### Resistance Gene Characterization

Each isolate showing ESBL phenotype was screened by PCR for *bla*_SHV_, *bla*_CTX–M_, and for CTX-M-1, 2, 8, and 9 subgroups (Ebrahimi et al., 2014, 2016a,b). All amplicons were purified by QIAquick Gel Extraction Kit (Qiagen, Hilden, Germany) and further characterized by sequencing (Macrogen Europe, Amsterdam, Netherlands). Sequences were analyzed by CLC Main Workbench (CLC Bio, Aarhus, Denmark).

To investigate the presence of plasmid-mediated colistin resistance genes *mcr-1*, *mcr-2*, *mcr-3*, *mcr-4*, and *mcr-5*, a multiplex PCR assay was used (Rebelo et al., 2018).

### Genetic Diversity and Relatedness of the Strains

We determined the different *E. coli* phylogenetic groups by the multiplex PCR method developed by Clermont et al. (2013). A multiplex PCR assay was performed to detect the presence of virulence factor genes characteristic for enterovirulent *E. coli* pathotypes (Persson et al., 2007). To identify the *E. coli* sequence type (ST) 131 clonal lineage and its members [clades A, B, C, and C subclades (C1-M27, C1-non-M27, and C2)], we used the multiplex PCR developed by Matsumura et al. (2017).

To analyze the epidemiological relationship, we used pulsed-field gel electrophoresis (PFGE) as previously described (Ebrahimi et al., 2014). The threshold for probable genetic relatedness was set to a similarity of >85%.

### Whole Genome Sequencing

Based on the results of the PFGE, 20 isolates were selected for whole genome sequencing (WGS) to represent major pulsotypes carried by birds as well as pulsotypes that contained both human and bird isolates to reveal possible connections. Genomic DNA was extracted using Zixpress-32 Bacterial DNA Extraction Kit on Zixpress-32 Automated Nucleic Acid Purification Instrument (Zinexts Life Science Corporation) following the manufacturer’s instructions. WGS was performed using Nextera XT DNA Library Preparation Kit followed by 150-bp single-end sequencing on Illumina NextSeq500 platform. FASTQ files were quality trimmed then assembled *de novo* using Velvet (v1.0.0.); these are available under BioProject ID PRJNA693168. ResFinder (Camacho et al., 2009; Bortolaia et al., 2020; Zankari et al., 2020), PlasmidFinder (Camacho et al., 2009; Carattoli et al., 2014), and VirulenceFinder (Joensen et al., 2014; Malberg Tetzschner et al., 2020) available from the Center for Genomic Epidemiology^1^ were used to identify resistance genes, plasmid replicon types, and virulence factors. Multi-locus sequence typing (MLST) and core genome MLST (cgMLST) were performed using SeqSphere + (Ridom, Münster, Germany) according to the “*E. coli* MLST Warwick v1.0” and “*E. coli* cgMLST” version 1.0 scheme.

## Results

### Occurrence and Characteristics of Extended-Spectrum Beta-Lactamase-Producing *E. coli* in Rooks

Extended-spectrum beta-lactamase-producing bacteria were carried by 37 (33%) of 112 sampled birds and a total of 43 isolates have been recovered, all of which were *E. coli*; six samples (8544, 8551, 8557, 8578, 8583, and HOR3) yielded two different morphologies and during further analysis they turned to be pheno- and genotypically different ESBL-producing *E. coli* isolates (Supplementary Figure 2). The predominant ESBL genes were *bla*_CTX–M–55_ (16/43) followed by *bla*_CTX–M–27_ (*n* = 15/43) (Supplementary Figure 1). Fluoroquinolone (17/43) and sulfonamide (23/43) resistance was frequent whereas all isolates were susceptible to aminoglycosides; 40% (17/43) of the isolates were susceptible to examined non-beta-lactam antibiotics including all *bla*_CTX–M–15_ producers. The majority of the isolates carried by birds belonged to commensal phylogroups A (2.3%, 1/43), B1 (51.2%, 22/43), and C (7%, 3/43), B1 being the dominant phylogroup. However, a high proportion (40%, 17/43) of rook isolates belonged to phylogroups associated with human disease, B2 (34.9%, 15/43) and D (4.7%, 2/43) (Supplementary Figure 1). Two of B2 CTX-M-27-producing *E. coli* isolates proved to belong to the pandemic ST131 clonal lineage, to the recently emerged C1-M27 subclone. In addition, 21% (9/43) of the isolates carried the intimin coding *eae* gene.

### Fecal Carriage Rate and Characteristics of Extended-Spectrum Beta-Lactamase-Producing *E. coli* in Humans

In 2,455 human fecal samples, 42 ESBL-producing *E. coli* were found corresponding to a fecal carriage rate of 1.7%. The dominant ESBL genotypes were *bla*_CTX–M–15_ (20/42) followed by *bla*_CTX–M–27_ (10/42) (Supplementary Figure 1). Resistance to fluoroquinolones (24/42), sulfonamides (29/42), amikacin (14/42), gentamicin (12/42), and to tobramycin (14/42) was common. Isolates of commensal phylogroups were more prevalent; four, three, eight, and eight isolates belonged to phylogroup A, B1, C, and E, but overall B2 (17/42) was the dominant phylogroup. Of the B2 isolates, two, one, one, and ten belonged to ST131 clade A, B, subclade C2, and subclade C1-M27, respectively.

### Characteristics of Extended-Spectrum Beta-Lactamase-Producing *E. coli* From Inpatients

The dominant ESBL genes were *bla*_CTX–M–15_ (18/42) and *bla*_CTX–M–27_ (13/42) (Supplementary Figure 1). As sole ESBL gene, *bla*_SHV–12_ was present in two isolates. Co-resistance rates were high; 60% (25/42) of isolates were resistant to fluoroquinolones, sulfonamides, and aminoglycosides, mostly the *bla*_CTX–M–15_ producers. The majority (74%, 31/42) of the isolates belonged to B2 phylogroup with high prevalence (62%, 26/42) of ST131 clones. Among ST131 isolates, one, nine, and 16 belonged to clade B, subclade C2, and subclade C1-M27, respectively. Curiously, three ST131 C1-M27 isolates were *bla*_CTX–M–15_ producers.

### Comparing the Characteristics of Rook, Human Fecal, and Human Clinical Isolates

In rooks, *bla*_CTX–M–55_ was the dominant ESBL gene while in humans it was *bla*_CTX–M–15_; *bla*_CTX–M–27_ was the second most common ESBL gene in all three isolate collections (Supplementary Figure 1). Group 2 and group 8 CTX-M types were not detected. Rook-derived isolates showed lower co-resistance rates to non-beta-lactam antibiotics than human clinical isolates. Isolates resistant to aminoglycosides, fluoroquinolones, and trimethoprim–sulfamethoxazole tend to carry CTX-M-1 group, particularly *bla*_CTX–M–15_, except for rooks where *bla*_CTX–M–15_ producers were susceptible; *bla*_CTX–M–27_ producers were resistant to fluoroquinolones and to trimethoprim–sulfamethoxazole but not to aminoglycosides. Carbapenem resistance was not detected in the recovered isolates. The majority of rook and human fecal isolates belonged to commensal phylogroups while B2 was dominant among human clinical isolates. The pandemic ST131 *E. coli* clonal lineage was present in isolates of rooks and humans with the dominance of C1-M27 subclade. All isolates were negative for plasmid-mediated colistin resistance genes tested.

### Molecular Epidemiology of the Isolates

Pulsed-field gel electrophoresis revealed that human clinical and fecal isolates clustered frequently together whereas the vast majority of rook isolates tended to cluster separately from human isolates (Supplementary Figure 2), although clusters containing both rook and human isolates were also found. Out of these, a cluster of eight human fecal, ten human clinical, and two rook isolates (EC069) was the largest, which belonged to the ST131 clone. Most isolates of the ST131 clone were grouped into three clusters (EC003, EC069, and EC380) although a few other ST131 isolates were randomly distributed. The EC003 cluster exclusively consisted of human clinical ST131 isolates while EC380 cluster contained human fecal ST131 isolates. In the EC069 cluster, the two corvid ST131 isolates showed PFGE profiles indistinguishable from human clinical and human fecal isolates. A smaller cluster of three human clinical, one human fecal, and one rook isolate (EC088) was also detected. In addition, clusters EC147 and EC183 each comprised one rook and one human fecal isolate.

**FIGURE 1:**
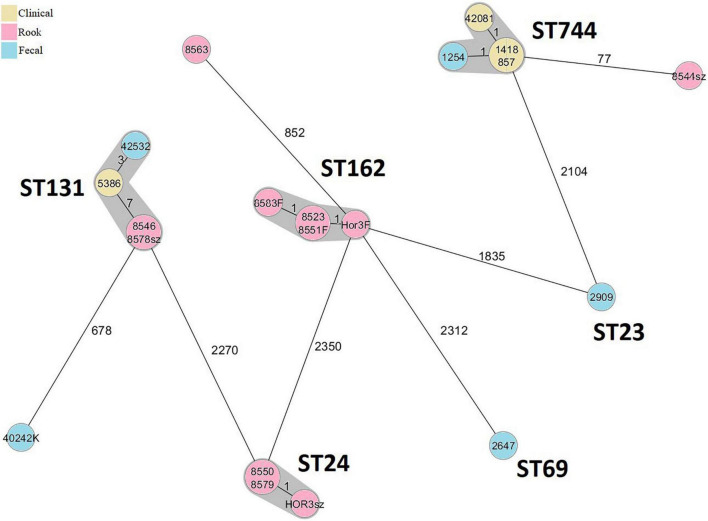
Minimum spanning tree based on cgMLST allelic profiles of 20 sequenced *E. coli* isolates. Each circle represents an allelic profile based on sequence analysis of 2,513 cgMLST target genes. The numbers on the connecting lines illustrate the numbers of target genes with different alleles. Closely related genotypes (<10 alleles difference) are shaded.

A total of eleven, four, and five of rook, human clinical, and human fecal isolates, respectively, were characterized by WGS (Table 1). The results of the cgMLST are shown in Figure 1. The relatedness of isolates in PFGE clusters of EC183 and EC399 were not supported by the results of the WGS (Table 1 and Supplementary Figure 2). Both ST24 and ST162 rook isolates were highly uniform genetically; distance based on allele presence was ≤1, although these isolates have been recovered from various birds in November and December. Human-derived ST744 isolates were closely related, but the rook one was relatively distant from this cluster; ST131 C1-M27 rook isolates were identical and in close connection with human strains (≤7 alleles) (Figure 1).

**TABLE 1:** Results of whole genome sequencing of the selected isolates.

PFGE	Strain source	ST	Resistance genes								Plasmid replicons
			*Bl*	Qui	Agl	Tri	Sul	Mac	Phe	Tet	
EC088	857 Clinical	744	*bla*CTX-M-1 *bla*CTX-M-14	gyrA p.S83L gyrA p.D87N parC p.A56T parC p.S80I	aadA5 aph(6)-Id aph(3″)-Ib	dfrA17	sul2	mdf(A)	catA1	tet(B)	IncFII IncI1-I IncQ1
	1254 Fecal	744	*bla*CTX-M-1 *bla*CTX-M-14	gyrA p.S83L gyrA p.D87N parC p.A56T	aadA5 aph(6)-Id aph(3″)-Ib	dfrA17	sul1 sul2	mdf(A)	catA1	tet(B)	IncFII IncI1-I IncQ1
	1418 Clinical	744	*bla*CTX-M-14	gyrA p.S83L gyrA p.D87N parC p.A56T parC p.S80I	aadA5 aph(6)-Id aph(3″)-Ib	dfrA17	sul1 sul2	mdf(A)	catA1	tet(B)	IncFII IncQ1
	8544sz Rook	744	*bla*CTX-M-55	qnrS1 gyrA p.S83L gyrA p.D87N parC p.A56T parC p.S80I	aadA5 aph(6)-Id aph(3″)-Ib	dfrA17	sul1	mdf(A) mph(A)	catA1	tet(A) tet(B)	IncFIA IncFIB IncFIC IncI1-I IncN
	42081 Clinical	744	*bla*CTX-M-14	gyrA p.S83L gyrA p.D87N parC p.A56T parC p.S80I	aadA5 aph(6)-Id aph(3″)-Ib	dfrA17	sul1 sul2		catA1	tet(B)	IncFII IncI1-I IncQ1
EC378	8579 Rook	24	*bla*CTX-M-27					mdf(A)			IncFIB IncFII
	8550 Rook	24	*bla*CTX-M-27					mdf(A)			IncFIB IncFII
	HOR3sz Rook	24	*bla*CTX-M-27					mdf(A)			IncFIB IncFII
EC069	5386 Clinical	131	*bla*CTX-M-27 *bla*TEM-1B	qnrS1 gyrA p.S83L gyrA p.D87N parC p.S80I parC p.E84V parE p.I529L	aadA5 aph(6)-Id aph(3″)-Ib	dfrA17	sul1 sul2	mdf(A) mph(A)		tet(A)	IncFIA IncFIB IncFII IncN
	42532 Fecal	131	*bla*CTX-M-27 *bla*TEM-1B	qnrS1 gyrA p.S83L gyrA p.D87N parC p.S80I parC p.E84V parE p.I529L	aadA5 aph(6)-Id aph(3″)-Ib	dfrA17	sul1 sul2	mdf(A)			IncFIA IncFIB IncFII IncN
	8578sz Rook	131	*bla*CTX-M-27	gyrA p.S83L gyrA p.D87N parC p.S80I parC p.E84V parE p.I529L	aadA5 aph(6)-Id aph(3″)-Ib	dfrA17	sul1 sul2	mdf(A) mph(A)		tet(A)	Col156 IncFIA IncFIB IncFII
	2647 Fecal	69	*bla*CTX-M-15	gyrA p.S83L		dfrA14		erm(B) mdf(A) mph(A)		tet(B)	Col156 IncFIA IncFIB IncFII IncX1
	8546 Rook	131	*bla*CTX-M-27	gyrA p.S83L gyrA p.D87N parC p.S80I parC p.E84V parE p.I529L	aadA5 aph(6)-Id aph(3″)-Ib	dfrA17	sul1 sul2	mdf(A) mph(A)		tet(A)	Col156 IncFIA IncFIB IncFII
EC183	40242k Fecal	131	*bla*CTX-M-15 *bla*TEM-1B	gyrA p.S83L parE p.I529L	aadA5 aph(6)-Id aph(3″)-Ib	dfrA17	sul1 sul2	mdf(A) mph(A)		tet(A)	Col156 IncFIB IncFII
	8563 Rook	162	*bla*CTX-M-1				sul2	mdf(A)		tet(A)	IncFIB IncFII IncI1-I IncX1
EC399	2909 Fecal	23	*bla*CTX-M-3					mdf(A)			IncFIB IncFIC IncI1-I
EC382	8523 Rook	162	*bla*CTX-M-55 *bla*TEM-1B	qnrS1	aph(6)-Id aph(3″)-Ib	dfrA7	sul1 sul2	mdf(A)		tet(A)	IncN lncQ1 p0111
	8583F Rook	162	*bla*CTX-M-55 *bla*TEM-1B	qnrS1	aph(6)-Id aph(3″)-Ib	dfrA7	sul1 sul2	mdf(A)		tet(A)	IncN lncQ1 p0111
	HOR3F Rook	162	*bla*CTX-M-55 *bla*TEM-1B	qnrS1	aph(6)-Id aph(3″)-Ib	dfrA7	sul1 sul2	mdf(A)		tet(A)	IncN lncQ1 p0111
	8551F Rook	162	*bla*CTX-M-55 *bla*TEM-1B	qnrS1	aph(6)-Id aph(3″)-Ib	dfrA7	sul1 sul2	mdf(A)		tet(A)	IncN lncQ1 p0111

*Bl, beta-lactam; Fq, fluoroquinolone; Agl, aminoglycoside; Tri, trimethoprim; Sul, sulfonamide; Mac, macrolide; Phe, phenicol; Tet, tetracycline.*

## Discussion

An increasing number of studies reported high prevalence of ESBL-producing *E. coli* in wild animals (Wang et al., 2017). Birds are the most studied hosts, where the most frequently found genes are *bla*_CTX–M–1_ and *bla*_CTX–M–15_ (Wang et al., 2017). Birds may serve as long distance vectors of strains/genes of human origin. Franklin’s gulls (*Leucophaeus pipixcan*) sampled in Chile frequently carried pandemic ST131 CTX-M-15-producing strains, which are highly prevalent in humans in the United States but rarely found in Chile (Báez et al., 2015). Similarly, *bla*_CTX–M–1_ and *bla*_CTX–M–15_ were previously dominant in rooks wintering in Europe (Loncaric et al., 2013; Jamborova et al., 2015). A few recent European studies also reported a low prevalence of *bla*_CTX–M–55_ and *bla*_CTX–M–24_ in rooks (Jamborova et al., 2015; Söderlund et al., 2019). Importantly, an earlier study from 2005 reported lack of ESBL producers in wintering rooks in the Czech Republic (Literak et al., 2007).

Rooks wintering in Hungary and in neighboring countries belong to a large population migrating from Russia and Western Asia, belonging to subspecies Western Rook (*Corvus frugilegus frugilegus*) with a breeding area stretching from the East European Plain to the West Siberian Plain. These populations migrate through the Black Sea–Mediterranean flyway and usually winter in European countries (Madge, 2020). The other rook subspecies Eastern Rook (*Corvus frugilegus pastinator*) nests in the Central Siberian Plateau and the Manchurian Plain and migrates to China and to Japan through the East Asian–Australasian flyway. On the border of West Siberian Plain and Central Siberian Plateau, there is a hybrid zone of the two subspecies, where birds may intermingle (Madge, 2020).

In the present work, *bla*_CTX–M–55_ and *bla*_CTX–M–27_ were predominant in rooks; *bla*_CTX–M–55_ is rarely reported in Europe from humans but highly prevalent in Southeast Asia (Lupo et al., 2018). It has been suggested that *bla*_CTX–M–55_ in humans in Asia arose from food animal sources, highlighting the importance of One Health (Bevan et al., 2017). Previously, *bla*_CTX–M–14_ and, to a lesser extent, *bla*_CTX–M–15_ were dominant ESBL genes in Asia; recently, *bla*_CTX–M–55_ emerged as the most common ESBL gene in human and animal isolates, while *bla*_CTX–M–27_ started to outcompete *bla*_CTX–M–14_ (Bevan et al., 2017). As *bla*_CTX–M–55_ is dominant in livestock in Asia (Bevan et al., 2017), and manure is often used to fertilize crop fields and may contain ESBL-producing *E. coli*, rooks foraging in these may acquire *bla*_CTX–M–55_ producers. This shift in the epidemiology of ESBL genes in Asia may be the cause of the alteration of ESBL genes in rooks as compared with earlier studies (Loncaric et al., 2013; Jamborova et al., 2015).

We hypothesized that Eastern Rooks acquire strains carrying resistance genes prevalent in animals and humans in China and transmit them to Western Rook individuals interacting with carrier Eastern Rooks in the hybrid area. Thus, intermingling rooks may become long-distance vectors mediating spread of strains/genes from Asia to Europe. Similarly, this may have been the route for clade C1-M27 described first in Japan in 2006 then in Korea in 2008 spreading since to Europe and to America (Matsumura et al., 2016). Similar spread routes of H5N1 avian influenza virus was reported extensively in different bird species (Gauthier-Clerc et al., 2007). High similarity between ST131 C1-M27 isolates of rook and human origin may also be explained by acquisition of these strains by rooks in Hungary, suggesting bidirectional transfer. Food importation may be another potential pathway where *bla*_CTX–M–55_ can spread from animals to humans toward Europe as exemplified by the dissemination mcr-1 resistance gene since a lot of poultry and pork are imported from China to Europe (Hasman et al., 2015) and the detection of these genes can often be traced back there (Lupo et al., 2018).

The majority of rook isolates of phylogroup B2 had indistinguishable macrorestriction profile (Supplementary Figure 2) identified as ST24 carrying *bla*_CTX–M–27_. ST24 was reported rarely, mainly from diarrheic rabbits, cattle, and humans (Moura et al., 2009; Xiong et al., 2012). All ST24 isolates carried the *eae* gene (Supplementary Table 1), which encodes a major virulence gene of enteropathogenic *E. coli* (EPEC); lack of the *bfp* gene indicates that these isolates are atypical EPEC strains, which are reported both from humans and animals (Moura et al., 2009). A study found that atypical EPEC strains of animal origin have potential to cause diarrhea in humans and revealed a close clonal relationship between human and animal isolates (Moura et al., 2009).

The main phylogroup was B1 among rook isolates, forming a large cluster belonging to ST162 and carrying *bla*_CTX–M–55_ (Supplementary Table 1). ST162 was found previously in rooks wintering in Europe with low occurrence (Loncaric et al., 2013; Jamborova et al., 2015). This emerging multiresistant *E. coli* lineage is now found worldwide colonizing different hosts including livestock, wild animals, humans, rivers, and sewage (Fuentes-Castillo et al., 2020). ST162 was reported from dairy cows with mastitis (Tahar et al., 2020) and from human clinical samples, even associated with *bla*_NDM–5_ in humans (Yoon et al., 2018). These associations raise the concern of dissemination of commensal multiresistant strains in human populations and the diffusion of the antibiotic resistance carried by these strains to other non-commensal, pathogenic strains (Zhuge et al., 2019). Moreover, ST162 *E. coli* recovered from poultry was identified as a highly virulent clone, despite belonging to phylogroup B1, capable of causing bloodstream infections and meningitis in animal models (Zhuge et al., 2019). In our study, ST162 and ST24 isolates seemed to have clonally expanded in rooks, which is notable as the clonal spread of ESBL-producing *E. coli* is scarcely documented in wild animals (Lupo et al., 2018).

Sequence types with human importance ST744 and ST131 C1-M27 were found seldom. ST744 is an international high-risk clone identified in our rook, human fecal, and clinical isolates (Table 1). ST744 carrying *bla*_CTX–M–55_ was previously reported from diseased pigs in the United States and from healthy and diseased bovines in France, from wastewater, birds of prey (Guenther et al., 2012; Lupo et al., 2018; Hayer et al., 2020) as well as from healthy and diseased companion animals, and humans (Tacão et al., 2017; Zhong et al., 2017; Zogg et al., 2018). Besides ESBLs, ST744 was sporadically associated with mcr-1, mcr-3, *bla*_KPC–3_, and *bla*_NDM_ genes from patients, healthy individuals, and livestock worldwide (Tacão et al., 2017; Zhong et al., 2017; Lupo et al., 2018; Zogg et al., 2018; Li et al., 2019). ST744 isolates of phylogroup A are not as virulent as those belonging to phylogroup B2 and D, but our ST744 rook isolate carried 15 virulence factors (Supplementary Table 1) commonly found in extraintestinal pathogenic *E. coli* (ExPEC) isolates.

Presence of key virulence genes (*iss*, *iroN*, *hlyF* and *ompT*, *iutA* and *cvaC*) and phylogroup A indicates that our rook ST744 is an avian pathogenic *E. coli* (APEC) strain, which is the main cause of avian colibacillosis (Johnson et al., 2008). Therefore, wild birds carrying APEC strains might pose a potential economic risk toward poultry; these genes are also frequently found among human ExPEC strains, raising the possibility of zoonotic transmission (Johnson et al., 2008; Zhuge et al., 2019). These suggest that ST744 may be a zoonotic strain capable of colonizing and infecting multiple host species including humans. Moreover, its potential to carry multiple plasmids predisposes it to be involved in transmission of resistance plasmids to other *E. coli* STs.

Subclade ST131 C1-M27 is associated with clonal spread in humans, and was also reported from great cormorants (*Phalacrocorax carbo*), mallards (*Anas platyrhynchos*) (Tausova et al., 2012), gulls (Zendri et al., 2020), companion animals, freshwater, and wastewater (Bevan et al., 2017). Similarly, it occurred in rooks and was also prevalent in human isolates in our study.

Although *bla*_CTX–M–15_ remained the predominant ESBL gene among asymptomatically carried human isolates in this study, the prevalence of ST131-CTX-M-15 *E. coli* was lower compared with earlier findings (Ebrahimi et al., 2014, 2016a,b), suggesting a slow replacement of C2 subclade carrying *bla*_CTX–M–15_ by the C1-M27 subclade (Merino et al., 2018). ST131 C1-M27 had a higher transmission rate than CTX-M-15-producing ST131 C2 (Merino et al., 2018). C1-M27 isolates often show lower co-resistance to other antimicrobial agents than C2 isolates (Jamborova et al., 2018), which may be advantageous in an antibiotic landscape dominated by beta-lactams as seen in many European countries including Hungary, and particularly the setting where this study was conducted (Tóth et al., 2019).

Both *bla*_CTX–M–27_ and *bl*a_CTX–M–55_ are associated with a wide range of plasmid replicons in animal isolates and certain plasmids showed epidemic spread in Asia in humans (Bevan et al., 2017; Lupo et al., 2018). Unlike *bla*_CTX–M–27_ associated with ST131 C1-M27, horizontal transmission is considered to be the main factor driving the dissemination of *bla*_CTX–M–55_ in China (Ho et al., 2013). In our work, *bla*_CTX–M–55_ have been associated with IncN replicon type (Table 1), which often harbors various ESBL genes but rarely *bla*_CTX–M–55_^2^. This association of *bla*_CTX–M–55_ with IncN plasmids carried by ST162 may open a new way to the dissemination of *bla*_CTX–M–55_ in and from Asia toward Europe by bird migration or vagrancy as it may have earlier happened in the case of ST131 C1-M27 (Matsumura et al., 2016).

In summary, increased carriage of ESBL-producing *E. coli* was found in rooks than reported in previous years. Despite the possibilities for contact, birds and humans shared a low proportion of genotypes. The presence of high-risk clones (ST131 and ST744), high prevalence of Asia-related ESBL genes (*bla*_CTX–M–55_ and *bla*_CTX–M–27_) together with the epidemiological history of ST131 C1-M27 clone suggests that rooks are among the potential vectors for the dissemination of antibiotic resistance genes and resistant strains.

## Data Availability Statement

The datasets presented in this study can be found in online repositories. The names of the repository/repositories and accession number(s) can be found in the article/Supplementary Material.

## Ethics Statement

Ethical review and approval was not required for the animal study because the birds were captured for the purpose of bird ringing. Bird ringing permission number 309 (Birdlife Hungary MME).

## Author Contributions

GK, LK, ÁL-K, and ÁT: conceptualization. BN, BB, and PG: data curation. BN, EK, KBl, and ÁT: formal analysis. BN, BB, IB, ID, and KBl: investigation. PG, LK, ÁL-K, ID, BN, LM, ÁT, and KBn: methodology. LK, ÁT, KBn, and GK: resources. EK, KBl, ID, and ÁT: software. ÁT and GK: supervision. PG, ÁL-K, ID, LM, ÁT, and KBn: validation. ID and ÁT: visualization. BN and GK: writing—original draft. BN, ÁT, and GK: writing—review and editing. All authors contributed to the article and approved the submitted version.

## Conflict of Interest

The authors declare that the research was conducted in the absence of any commercial or financial relationships that could be construed as a potential conflict of interest.

## Publisher’s Note

All claims expressed in this article are solely those of the authors and do not necessarily represent those of their affiliated organizations, or those of the publisher, the editors and the reviewers. Any product that may be evaluated in this article, or claim that may be made by its manufacturer, is not guaranteed or endorsed by the publisher.

## Footnotes

1
http://www.genomicepidemiology.org

2
https://pubmlst.org/organisms/plasmid-mlst
