# Supplementary material for: Comparison of Extended-Spectrum Beta-Lactamase-Producing Escherichia coli Isolates From Rooks (Corvus frugilegus) and Contemporary Human-Derived Strains: A One Health Perspective
Source: Front Microbiol. 2022 Jan 13;12:785411. doi: 10.3389/fmicb.2021.785411 (PMC8792927; doi:10.3389/fmicb.2021.785411)
Supplement: Supplementary file 1 [file Data_Sheet_1.docx]

Supplement 1: Distribution of CTX-M genotypes among different E. coli phylogroups.

Filogroup F was not detected.

*Two clinical isolates carried bla_SHV-12_ and not counted on this figure.
