# Supplementary material for: Comparison of Extended-Spectrum Beta-Lactamase-Producing Escherichia coli Isolates From Rooks (Corvus frugilegus) and Contemporary Human-Derived Strains: A One Health Perspective
Source: Front Microbiol. 2022 Jan 13;12:785411. doi: 10.3389/fmicb.2021.785411 (PMC8792927; doi:10.3389/fmicb.2021.785411)
Supplement: Supplementary file 2 [file Data_Sheet_2.pdf]

e (Opt:1.00%) (Tol 1.0%-2.0%) (H>0.0% S>0.0%) [0.0%-100.0%]
bsiepi

klebsiepi

Supplement 2: Macrorestriction profile of the *Escherichia coli* isolates. Isolates with frames were selected for whole genome sequencing.

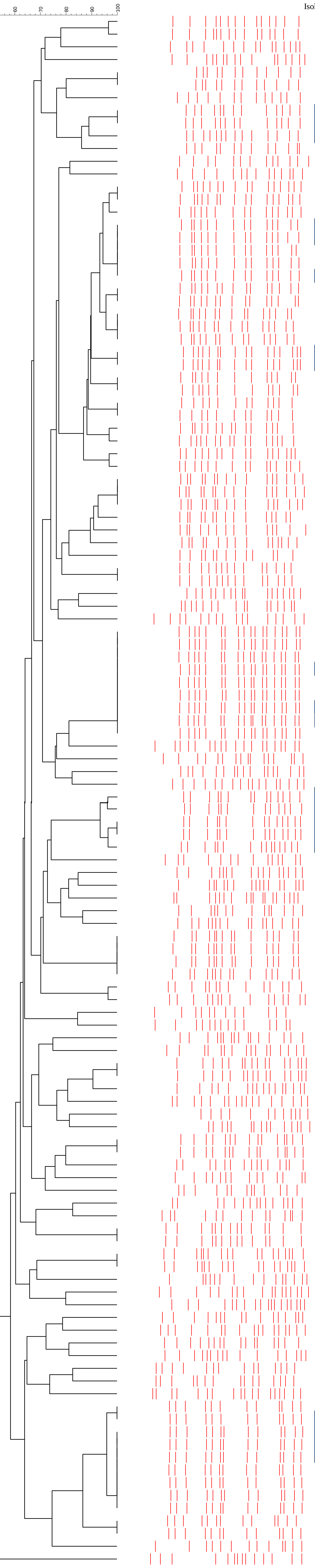

Isolate number	Pulsotype	ESBL gene	Source	Phylogroup
8569	EC390	CTX-M-1	corvid	B1
8570	EC390	CTX-M-1	corvid	B1
35051	EC389	CTX-M-15	faeces	B1
32416	.EC383	CTX-M-15	sputum	B2
31638	EC379	CTX-M-15	cervix	B2
35840/2	EC379	CTX-M-15	throat	B2
36884/1	EC335	CTX-M-65	bronchus	B1
40242 K	EC183	CTX-M-15	faeces	B2
8563	EC183	CTX-M-1	corvid	B1
2929	EC399	CTX-M-15	faeces	E
284 R	EC400	CTX-M-15	faeces	B2
6745	EC397	CTX-M-15	urine	B2
8573	EC275	CTX-M-1	corvid	B1
40531	EC069	CTX-M-27	faeces	B2
3699	EC069	CTX-M-27	bronchus	B2
1158	EC069	CTX-M-27	faeces	B2
8578 SZ	EC069	CTX-M-27	corvid	B2
2647	EC069	CTX-M-15	faeces	D
39967	EC069	CTX-M-15	urine	B2
6771	EC069	CTX-M-27	faeces	B2
8546	EC069	CTX-M-27	corvid	B2
42825	EC069	CTX-M-27	faeces	B2
35797	EC069	CTX-M-27	urine	B2
1713/1	EC069	CTX-M-15	abscess	B2
37293	EC069	CTX-M-27	cervix	B2
645 K	EC069	CTX-M-27	faeces	B2
5386	EC069	CTX-M-27	urine	B2
42532	EC069	CTX-M-27	faeces	B2
32433	EC069	CTX-M-27	urine	B2
7362	EC069	CTX-M-27	ascites	B2
2236	EC368	CTX-M-15	wound	B2
1751/1	EC368	CTX-M-9	urine	B2
36482/2	EC069	CTX-M-27	urine	B2
37493	EC069	CTX-M-27	urine	B2
7031	EC249	CTX-M-27	bronchus	B2
7240	EC249	CTX-M-27	urine	B2
5342	EC003	CTX-M-15	urine	B2
5396	EC003	CTX-M-15	abscess	B2
37328	EC003	CTX-M-15	urine	B2
1517/1	EC003	CTX-M-15	urine	B2
2037/1	EC003	CTX-M-15	urine	B2
37931/1	EC003	CTX-M-15	sputum	B2
38905	EC402	CTX-M-15	bronchus	B2
40607	EC364	CTX-M-15	faeces	B2
40002	EC364	CTX-M-15	urine	B2
33762/1	EC401	CTX-M-3	wound	B2
41675	EC427	CTX-M-15	blood	B2
35051	EC408	CTX-M-15	faeces	B1
8557-2E	EC378	CTX-M-27	corvid	B2
8583 SZ	EC378	CTX-M-14	corvid	B2
8551 SZ	EC378	CTX-M-27	corvid	B2
8579	EC378	CTX-M-27	corvid	B2
8560	EC378	CTX-M-27	corvid	B2
8563 SZ	EC378	CTX-M-27	corvid	B2
8550	EC378	CTX-M-27	corvid	B2
HÖR3 SZ	EC378	CTX-M-27	corvid	B2
8572	EC378	CTX-M-27	corvid	B2
8548	EC396	CTX-M-15, CTX-M-27	corvid	B2
504 K	EC409	CTX-M-1	faeces	A
738	EC398	CTX-M-15	faeces	B2
31935	EC392	CTX-M-32	wound	B2
8544 SZ	EC088	CTX-M-55	corvid	A
42081	EC088	CTX-M-14	urine	A
1418/1	EC088	CTX-M-14	throat	A
1254	EC088	CTX-M-1, CTX-M-14	faeces	A
857	EC088	CTX-M-1, CTX-M-14	urine	A
41914	EC403	CTX-M-15	faeces	C
2908	EC405	CTX-M-15	faeces	A
8582	EC407	CTX-M-15	corvid	C
1992	EC404	CTX-M-1	faeces	C
6567	EC426	CTX-M-32	faeces	C
5551	EC406	SHV-12	abscess	C
33771	EC380	CTX-M-27	urine	B2
34588	EC380	CTX-M-27	faeces	B2
34533	EC380	CTX-M-27	faeces	B2
34727	EC380	CTX-M-27	faeces	B2
8767X3	EC147	CTX-M-55	corvid	B2
418	EC147	CTX-M-27	faeces	B2
8576 X1	EC410	CTX-M-15	corvid	B1
8567	EC411	CTX-M-3, CTX-M-27	corvid	B2
2977	EC385	CTX-M-15	faeces	B1
42352	EC395	CTX-M-1	faeces	A
35515 R	EC376	CTX-M-15	faeces	C
35515	EC376	CTX-M-15	faeces	C
6464	EC388	CTX-M-15	faeces	C
2130	EC416	CTX-M-14	faeces	E
42398	EC391	CTX-M-15	urine	C
35740 K	EC377	CTX-M-15	faeces	C
7780	EC413	CTX-M-15	urine	B2
37497/1	EC413	CTX-M-15	wound	B2
5559 R	EC387	CTX-M-15	faeces	B2
218 K	EC414	CTX-M-3	faeces	B2
8521	EC384	CTX-M-55	corvid	B1
8571	EC393	CTX-M-32	corvid	B1
42916	EC394	SHV-12	urine	B1
8543	EC375	CTX-M-1	corvid	C
8540	EC375	CTX-M-1	corvid	C
8556X2	EC381	CTX-M-27	corvid	D
8557-1	EC381	CTX-M-27	corvid	D
559	EC386	CTX-M-3	faeces	C
36922/1	EC418	CTX-M-15	bronchus	A
6041	EC422	CTX-M-15	faeces	A
33720	EC421	CTX-M-27	urine	B2
41126 K	EC372	CTX-M-15	faeces	D
5563	EC420	CTX-M-15	faeces	E
38042	EC412	CTX-M-3	faeces	B1
6071	EC417	CTX-M-3	faeces	E
1148 R	EC099	CTX-M-15	faeces	E
6579	EC419	CTX-M-15	faeces	E
8527	EC382	CTX-M-55	corvid	B1
8523	EC382	CTX-M-55	corvid	B1
8583 F	EC382	CTX-M-55	corvid	B1
HÖR3 F	EC382	CTX-M-55	corvid	B1
8551 F	EC382	CTX-M-55	corvid	B1
8544 F	EC382	CTX-M-55	corvid	B1
8529	EC382	CTX-M-55	corvid	B1
8558	EC382	CTX-M-55	corvid	B1
8532 SZ	EC382	CTX-M-55	corvid	B1
8522X3	EC382	CTX-M-55	corvid	B1
8578 F	EC382	CTX-M-55	corvid	B1
41626/2	EC425	CTX-M-1	wound	B1
284	EC423	CTX-M-15	faeces	B2
