# Supplementary material for: Comparison of Extended-Spectrum Beta-Lactamase-Producing Escherichia coli Isolates From Rooks (Corvus frugilegus) and Contemporary Human-Derived Strains: A One Health Perspective
Source: Front Microbiol. 2022 Jan 13;12:785411. doi: 10.3389/fmicb.2021.785411 (PMC8792927; doi:10.3389/fmicb.2021.785411)
Supplement: Supplementary file 3 [file Data_Sheet_3.docx]

| **Supplement 3: Virulence genes carried by the whole genome sequenced isolates.** | | | |
| --- | --- | --- | --- |
| PFGE Pulsotype | Strain | Virulence gene | Function |
| EC088 | 857  Clinical | *gad* | Glutamate decarboxylase |
|  |  | *terC* | Tellurium ion resistance protein |
|  |  | *traT* | Outer membrane protein complement resistance |
|  | 1254 Faecal | *gad* | Glutamate decarboxylase |
|  |  | *terC* | Tellurium ion resistance protein |
|  |  | *traT* | Outer membrane protein complement resistance |
|  | 1418/1 Clinical | *gad* | Glutamate decarboxylase |
|  |  | *terC* | Tellurium ion resistance protein |
|  |  | *traT* | Outer membrane protein complement resistance |
|  | 8544sz Rook | *cia* | Colicin ia |
|  |  | *cma* | Colicin M |
|  |  | *cvaC* | Microcin C |
|  |  | *etsC* | Putative type I secretion outer membrane protein |
|  |  | *gad* | Glutamate decarboxylase |
|  |  | *hlyf* | Hemolysin F |
|  |  | *iss* | Increased serum survival |
|  |  | *iroN* | Enterobactin siderophore receptor protein |
|  |  | *iucC* | Aerobactin synthetase |
|  |  | *iutA* | Ferric aerobactin receptor |
|  |  | *mchF* | ABC transporter protein MchF |
|  |  | *ompT* | Outer membrane protease (protein protease 7) |
|  |  | *sitA* | Iron transport protein |
|  |  | *terC* | Tellurium ion resistance protein |
|  |  | *traT* | Outer membrane protein complement resistance |
|  | 42081 Clinical | *cia* | Colicin ia |
|  |  | *gad* | Glutamate decarboxylase |
|  |  | *terC* | Tellurium ion resistance protein |
|  |  | *traT* | Outer membrane protein complement resistance |
| EC378 | 8579 Rook | *chuA* | Outer membrane hemin receptor |
|  |  | *cif* | Type III secreted effector |
|  |  | *eae* | Intimin |
|  |  | *espA* | Type III secretions system |
|  |  | *espC* | EPEC secreted protein C |
|  |  | *espF* | Type III secretion system |
|  |  | *espJ* | Prophage-encoded type III secretion system effector |
|  |  | *nleB* | Non-LEE encoded effector B |
|  |  | *ompT* | Outer membrane protease (protein protease 7) |
|  |  | *terC* | Tellurium ion resistance protein |
|  |  | *tir* | Translocated intimin receptor protein |
|  |  | *toxB* | Toxin B |
|  |  | *yfcV* | Fimbrial protein |
|  | 8550 Rook | *chuA* | Outer membrane hemin receptor |
|  |  | *cif* | Type III secreted effector |
|  |  | *eae* | Intimin |
|  |  | *espA* | Type III secretions system |
|  |  | *espC* | EPEC secreted protein C |
|  |  | *espF* | Type III secretion system |
|  |  | *espJ* | Prophage-encoded type III secretion system effector |
|  |  | *gad* | Glutamate decarboxylase |
|  |  | *nleB* | Non-LEE encoded effector B |
|  |  | *ompT* | Outer membrane protease (protein protease 7) |
|  |  | *terC* | Tellurium ion resistance protein |
|  |  | *tir* | Translocated intimin receptor protein |
|  |  | *toxB* | Toxin B |
|  |  | *yfcV* | Fimbrial protein |
|  | HOR3SZ Rook | *chuA* | Outer membrane hemin receptor |
|  |  | *cif* | Type III secreted effector |
|  |  | *eae* | Intimin |
|  |  | *espA* | Type III secretions system |
|  |  | *espC* | EPEC secreted protein C |
|  |  | *espF* | Type III secretion system |
|  |  | *espJ* | Prophage-encoded type III secretion system effector |
|  |  | *nleB* | Non-LEE encoded effector B |
|  |  | *ompT* | Outer membrane protease (protein protease 7) |
|  |  | *terC* | Tellurium ion resistance protein |
|  |  | *tir* | Translocated intimin receptor protein |
|  |  | *toxB* | Toxin B |
|  |  | *yfcV* | Fimbrial protein |
| EC069 | 5386 Clinical | *chuA* | Outer membrane hemin receptor |
|  |  | *fyuA* | Siderophore receptor |
|  |  | *iha* | Adherence protein |
|  |  | *irp2* | High molecular weight protein 2 non-ribosomal peptide synthetase |
|  |  | *iss* | Increased serum survival |
|  |  | *iucC* | Aerobactin synthetase |
|  |  | *iutA* | Ferric aerobactin receptor |
|  |  | *kpsE* | Capsule polysaccharide export inner-membrane protein |
|  |  | *kpsMII_K5* | Polysialic acid transport protein; Group 2 capsule |
|  |  | *ompT* | Outer membrane protease (protein protease 7) |
|  |  | *papA_F43* | Major pilin subunit F43 |
|  |  | *sat* | Secreted autotransporter toxin |
|  |  | *sitA* | Iron transport protein |
|  |  | *terC* | Tellurium ion resistance protein |
|  |  | *traT* | Outer membrane protein complement resistance |
|  |  | *usp* | Uropathogenic specific protein |
|  |  | *yfcV* | Fimbrial protein |
|  | 42532 Faecal | *chuA* | Outer membrane hemin receptor |
|  |  | *fyuA* | Siderophore receptor |
|  |  | *iha* | Adherence protein |
|  |  | *irp2* | High molecular weight protein 2 non-ribosomal peptide synthetase |
|  |  | *iss* | Increased serum survival |
|  |  | *iucC* | Aerobactin synthetase |
|  |  | *iutA* | Ferric aerobactin receptor |
|  |  | *kpsE* | Capsule polysaccharide export inner-membrane protein |
|  |  | *kpsMII_K5* | Polysialic acid transport protein; Group 2 capsule |
|  |  | *ompT* | Outer membrane protease (protein protease 7) |
|  |  | *papA_F43* | Major pilin subunit F43 |
|  |  | *sat* | Secreted autotransporter toxin |
|  |  | *sitA* | Iron transport protein |
|  |  | *terC* | Tellurium ion resistance protein |
|  |  | *traT* | Outer membrane protein complement resistance |
|  |  | *usp* | Uropathogenic specific protein |
|  |  | *yfcV* | Fimbrial protein |
|  | 8578sz Rook | *chuA* | Outer membrane hemin receptor |
|  |  | *fyuA* | Siderophore receptor |
|  |  | *iha* | Adherence protein |
|  |  | *irp2* | High molecular weight protein 2 non-ribosomal peptide synthetase |
|  |  | *iss* | Increased serum survival |
|  |  | *iucC* | Aerobactin synthetase |
|  |  | *iutA* | Ferric aerobactin receptor |
|  |  | *kpsE* | Capsule polysaccharide export inner-membrane protein |
|  |  | *kpsMII_K5* | Polysialic acid transport protein; Group 2 capsule |
|  |  | *ompT* | Outer membrane protease (protein protease 7) |
|  |  | *papA_F43* | Major pilin subunit F43 |
|  |  | *sat* | Secreted autotransporter toxin |
|  |  | *senB* | Plasmid-encoded enterotoxin |
|  |  | *sitA* | Iron transport protein |
|  |  | *terC* | Tellurium ion resistance protein |
|  |  | *traT* | Outer membrane protein complement resistance |
|  |  | *usp* | Uropathogenic specific protein |
|  |  | *yfcV* | Fimbrial protein |
|  | 2647 Faecal | *air* | Enteroaggregative immunoglobulin repeat protein |
|  |  | *chuA* | Outer membrane hemin receptor |
|  |  | *eilA* | Salmonella HilA homolog |
|  |  | *fyuA* | Siderophore receptor |
|  |  | *iha* | Adherence protein |
|  |  | *irp2* | High molecular weight protein 2 non-ribosomal peptide synthetase |
|  |  | *iss* | Increased serum survival |
|  |  | *iucC* | Aerobactin synthetase |
|  |  | *iutA* | Ferric aerobactin receptor |
|  |  | *kpsE* | Capsule polysaccharide export inner-membrane protein |
|  |  | *kpsMII* | Polysialic acid transport protein; Group 2 capsule |
|  |  | *lpfA* | Long polar fimbriae |
|  |  | *mcbA* | Bacteriocin microcin B17 |
|  |  | *neuC* | Polysialic acid capsule biosynthesis protein |
|  |  | *ompT* | Outer membrane protease (protein protease 7) |
|  |  | *sat* | Secreted autotransporter toxin |
|  |  | *sitA* | Iron transport protein |
|  |  | *terC* | Tellurium ion resistance protein |
|  |  | *traT* | Outer membrane protein complement resistance |
|  | 8546 Rook | *chuA* | Outer membrane hemin receptor |
|  |  | *fyuA* | Siderophore receptor |
|  |  | *iha* | Adherence protein |
|  |  | *irp2* | High molecular weight protein 2 non-ribosomal peptide synthetase |
|  |  | *iss* | Increased serum survival |
|  |  | *iucC* | Aerobactin synthetase |
|  |  | *iutA* | Ferric aerobactin receptor |
|  |  | *kpsE* | Capsule polysaccharide export inner-membrane protein |
|  |  | *kpsMII_K5* | Polysialic acid transport protein; Group 2 capsule |
|  |  | *ompT* | Outer membrane protease (protein protease 7) |
|  |  | *papA_F43* | Major pilin subunit F43 |
|  |  | *sat* | Secreted autotransporter toxin |
|  |  | *senB* | Plasmid-encoded enterotoxin |
|  |  | *sitA* | Iron transport protein |
|  |  | *terC* | Tellurium ion resistance protein |
|  |  | *traT* | Outer membrane protein complement resistance |
|  |  | *usp* | Uropathogenic specific protein |
|  |  | *yfcV* | Fimbrial protein |
| EC183 | 40242k Faecal | *chuA* | Outer membrane hemin receptor |
|  |  | *fyuA* | Siderophore receptor |
|  |  | *gad* | Glutamate decarboxylase |
|  |  | *iha* | Adherence protein |
|  |  | *irp2* | High molecular weight protein 2 non-ribosomal peptide synthetase |
|  |  | *iucC* | Aerobactin synthetase |
|  |  | *iutA* | Ferric aerobactin receptor |
|  |  | *kpsE* | Capsule polysaccharide export inner-membrane protein |
|  |  | *kpsMII_K5* | Polysialic acid transport protein; Group 2 capsule |
|  |  | *mcbA* | Bacteriocin microcin B17 |
|  |  | *ompT* | Outer membrane protease (protein protease 7) |
|  |  | *papA_F43* | Major pilin subunit F43 |
|  |  | *sat* | Secreted autotransporter toxin |
|  |  | *senB* | Plasmid-encoded enterotoxin |
|  |  | *sitA* | Iron transport protein |
|  |  | *terC* | Tellurium ion resistance protein |
|  |  | *traT* | Outer membrane protein complement resistance |
|  |  | *usp* | Uropathogenic specific protein |
|  |  | *yfcV* | Fimbrial protein |
|  | 8563 Rook | *cia* | Colicin ia |
|  |  | *cvaC* | Microcin C |
|  |  | *etsC* | Putative type I secretion outer membrane protein |
|  |  | *gad* | Glutamate decarboxylase |
|  |  | *hlyF* | Hemolysin F |
|  |  | *iroN* | Enterobactin siderophore receptor protein |
|  |  | *iss* | Increased serum survival |
|  |  | *lpfA* | Long polar fimbriae |
|  |  | *mchF* | ABC transporter protein MchF |
|  |  | *ompT* | Outer membrane protease (protein protease 7) |
|  |  | *sitA* | Iron transport protein |
|  |  | *terC* | Tellurium ion resistance protein |
|  |  | *traT* | Outer membrane protein complement resistance |
| EC309 | 2909 Faecal | *cvaC* | Microcin C |
|  |  | *etsC* | Putative type I secretion outer membrane protein |
|  |  | *fyuA* | Siderophore receptor |
|  |  | *gad* | Glutamate decarboxylase |
|  |  | *hlyF* | Hemolysin F |
|  |  | *hra* | Heat-resistant agglutinin |
|  |  | *iroN* | Enterobactin siderophore receptor protein |
|  |  | *irp2* | High molecular weight protein 2 non-ribosomal peptide synthetase |
|  |  | *iss* | Increased serum survival |
|  |  | *iucC* | Aerobactin synthetase |
|  |  | *iutA* | Ferric aerobactin receptor |
|  |  | *lpfA* | Long polar fimbriae |
|  |  | *mchF* | ABC transporter protein MchF |
|  |  | *ompT* | Outer membrane protease (protein protease 7) |
|  |  | *papA_F48* | Major pilin subunit F48 |
|  |  | *papC* | Outer membrane usher P fimbriae |
|  |  | *sitA* | Iron transport protein |
|  |  | *terC* | Tellurium ion resistance protein |
|  |  | *traT* | Outer membrane protein complement resistance |
|  |  | *tsh* | Temperature-sensitive hemagglutinin |
| EC382 | 8523 Rook | *cba* | Colicin B |
|  |  | *gad* | Glutamate decarboxylase |
|  |  | *lpfA* | Long polar fimbriae |
|  |  | *neuC* | Polysialic acid capsule biosynthesis protein |
|  |  | *terC* | Tellurium ion resistance protein |
|  | 8583F Rook | *cba* | Colicin B |
|  |  | *gad* | Glutamate decarboxylase |
|  |  | *lpfA* | Long polar fimbriae |
|  |  | *neuC* | Polysialic acid capsule biosynthesis protein |
|  |  | *terC* | Tellurium ion resistance protein |
|  | HOR3F Rook | *gad* | Glutamate decarboxylase |
|  |  | *lpfA* | Long polar fimbriae |
|  |  | *neuC* | Polysialic acid capsule biosynthesis protein |
|  |  | *terC* | Tellurium ion resistance protein |
|  | 8551F Rook | *cba* | Colicin B |
|  |  | *gad* | Glutamate decarboxylase |
|  |  | *lpfA* | Long polar fimbriae |
|  |  | *neuC* | Polysialic acid capsule biosynthesis protein |
|  |  | *terC* | Tellurium ion resistance protein |
